# Deposition of Biogenic Iron Minerals in a Methane Oxidizing Microbial Mat

**DOI:** 10.1155/2013/102972

**Published:** 2013-06-13

**Authors:** Christoph Wrede, Sebastian Kokoschka, Anne Dreier, Christina Heller, Joachim Reitner, Michael Hoppert

**Affiliations:** ^1^Institute of Microbiology and Genetics, Georg-August-University, Grisebachstr. 8, 37077 Göttingen, Germany; ^2^Hannover Medical School, Institute of Functional and Applied Anatomy, Carl-Neuberg-Str. 1, 30625 Hannover, Germany; ^3^Courant Centre Geobiology, Georg-August-University, Goldschmidtstr. 3, 37077 Göttingen, Germany; ^4^Geoscience Centre Göttingen, Georg-August-University, Goldschmidtstr. 3, 37077 Göttingen, Germany; ^5^Federal Institute for Geosciences and Natural Resources, Stilleweg 2, 30655 Hannover, Germany

## Abstract

The syntrophic community between anaerobic methanotrophic archaea and sulfate reducing bacteria forms thick, black layers within multi-layered microbial mats in chimney-like carbonate concretions of methane seeps located in the Black Sea Crimean shelf. The microbial consortium conducts anaerobic oxidation of methane, which leads to the formation of mainly two biomineral by-products, calcium carbonates and iron sulfides, building up these chimneys. Iron sulfides are generated by the microbial reduction of oxidized sulfur compounds in the microbial mats. Here we show that sulfate reducing bacteria deposit biogenic iron sulfides extra- and intracellularly, the latter in magnetosome-like chains. These chains appear to be stable after cell lysis and tend to attach to cell debris within the microbial mat. The particles may be important nuclei for larger iron sulfide mineral aggregates.

## 1. Introduction 

Frequently, biofilm formation in marine and freshwater systems is accompanied by precipitation of minerals. These minerals are also structurally integrative parts of the microbial biofilm [1]. In most cases, mineral precipitates are deposited in close contact to and in interaction with organic macromolecules, that is, carbohydrates and/or proteins [2]. Formation of a biomineral in a microbial biofilm may be detrimental to the organisms which is mainly due to the enclosure of the living biomass by mineral precipitates. However, also positive effects, for example, when lithified precipitates provide a matrix or scaffold for the microbial biomass, may be expected. It has also been considered that beneficial effects predominate, for example, when biominerals act as chemical filters or shield UV radiation [3]. It is known that, in certain cases, biological macromolecules influence solubility of minerals (e.g., by buffering the aqueous environment or by chelating ions) and may direct the formation of a mineral matrix in a more or less specific way. As a consequence, the shape of biomineral deposits varies considerably at narrow scales and seemingly similar environmental conditions [4].

Mineral deposits caused by the activity of microorganisms are mostly based on either carbonates or silicates [4]. These mineral phases are regularly intermixed with other organic or mineralic compounds (overviews in [2, 5]). A special case of these organomineral precipitations is microbialite formation during anaerobic oxidation of methane (AOM). AOM is conducted by various groups of archaea in a metabolic pathway reverting methanogenesis [6]. Mostly, sulfate reducing bacteria (SRB) participate in AOM [7–9]. The role of SRB is still not fully understood, though is generally accepted that, along with the oxidation of methane, sulfate is reduced: CH_4_ + SO_4_^2−^ → HCO_3_^−^ + HS^−^ + H_2_O [8, 10]. As a result, carbonate phases (calcite and aragonite) and iron sulfides are generated as byproducts of the metabolic process. 

It is is known that AOM occurs worldwide in anoxic sediments when methane and electrons acceptors are available (e.g., [11]). The formation of large (several centimeters and bigger) carbonate concretions depends on high methane concentrations under hydrostatic pressure and on the presence of sulfate [8, 9, 12]. In the anaerobic water column of the Black Sea, huge carbonate concretions have been observed at the Crimean shelf [8]. The carbonate buildups may be considered as highly porous “fixed bed” bioreactors, allowing the percolation of methane and the exchange of sea water. The outer and inner surfaces of these carbonate buildups are covered by complex microbial mats, primarily formed by the organisms involved in AOM. 

In previous investigations, distinct layers in these microbial mats were discriminated. On the surface, exposed to the sea water, a black layer consists mainly of aggregates between methane-oxidizing archaea of the ANME-2 group and sulfate reducing bacteria (SRB). SRB of this mat type often exhibit intracytoplasmic magnetosome-like chains of greigite precipitations [8, 13, 14]. Our results imply that greigite magnetosomes are one sink for (otherwise toxic) sulfides. These particles were found inside SRB but were also present in the extracellular matrix of the biofilm.

## 2. Materials and Methods

Microbial mat samples were collected in 2001 during a cruise with the Russian R/V Professor Logachev in the methane seep area located in the GHOSTDABS field (Black Sea north east the Crimean shelf). These samples have already been subjected to extended geochemical and structural analyses [9, 14]. Specific antibodies, directed against methyl-coenzyme M reductase (MCR), the key enzyme of (reverse) methanogenesis, were generated after purification of MCR as essentially described according to [15] by immunization of rabbits following established protocols (e. g., [16] and, the references therein). Specificity of the antibody was extensively studied for methanogenic archaea and reverse methanogens as already described [14, 16, 17].

For microscopic analyses, the samples were chemically fixed in a 4.0% (v/v) aqueous formaldehyde solution (from a 10%, w/v, stock solution, pH 8.0, freshly prepared from paraformaldehyde) and stored in 100 mM PBS (phosphate buffered saline, pH 7.0) at 4°C until further use. The material was then washed several times in PBS and cut to small fragments of about 200 *μ*L volume. Samples were then chemically fixed in a 0.5% (v/v) glutardialdehyde solution (in 100 mM PBS) for 2 h. The samples were then processed as described [18], for electron and light microscopy, and finally cut in ultrathin or semithin sections of either 100–300 nm or 1 *μ*m in thickness. Semithin sections were transferred, with the aid of a transfer loop, on microscope slides, and ultrathin sections were picked up with Formvar-coated grids. For light microscopy, the sections were treated either with an anti-MCR antibody or with the lectin concanavalin A (ConA) coupled to fluorescent marker molecules (Sigma-Aldrich, Deisenhofen, Germany) as described [18]. The lectin ConA IV coupled to Alexa Fluor 546 as fluorescence marker (Molecular Probes, Eugene, OR, USA) was used in 1/1000 working concentration dilutions in PBS supplemented with 1 mM CaCl_2_ and MnCl_2_ (lectin buffer). The sections were mounted on glass slides by heat fixation at 60°C for 15 min and then incubated for 30 min at room temperature. After this, the lectin dilution was soaked off, and the sections were briefly rinsed in pure lectin buffer and covered with coverslips. For immunofluorescence microscopy, the heat-fixed semithin sections were incubated with the antiserum (dil. 1/1000 with PBS, pH 7.5) for 2 h. The sections were rinsed three times in 100 *μ*L drops of PBS (supplemented with 0.01% Tween 20) and incubated with a secondary goat anti-rabbit antibody, coupled to Alexa Fluor 546 fluorescent dye (Molecular Probes, Eugene, OR, USA), diluted 1 : 250 [14]. The rinsing steps were repeated. Fluorescence microscopy was performed with an Axio Scope light microscope using filter set 43 (BP: 545/25, FT 570, LP: 605/70) and the AxioVision software package (Zeiss, Göttingen, Germany). For comparison, phase contrast images were taken and were digitally merged with fluorescence images.

For transmission electron microscopy, ultrathin sections obtained from five distinct samples were mounted on Formvar-coated 300 mesh specimen grids. Immunolocalization with antibodies directed against MCR was performed as described. Mounted sections were stained with phosphotungstic acid (3%, w/v), if not stated otherwise [14]. Electron microscopy was performed in a Zeiss EM 902 transmission electron microscope (Zeiss, Oberkochen, Germany), equipped with a eucentric goniometer stage. Images were recorded with a 1 KB digital camera. Detection and enhancement of colloidal gold markers in digitized electron micrographs were performed as described [19]. Electron energy loss spectroscopy (EELS) of iron-containing particles was performed essentially as described in [20], with the aid of the analysis V software package (Olympus-SIS, Münster, Germany). Electron energy loss was measured between 655 eV and 751 eV. The L2 (720.6 eV) and L3 (708.0 eV) edges, observed for pure iron minerals, were represented by one broad peak at approximately 715 eV of the deposits in embedded and ultrathin sectioned biofilms. 

Goniometry was performed by tilting 300 nm sections ±60 degrees with 1-degree increments. Tomograms were performed with the EM3D 2.0 software package (Department of Neurobiology, Stanford University [21]).

## 3. Results and Discussion

**Figure 1:**
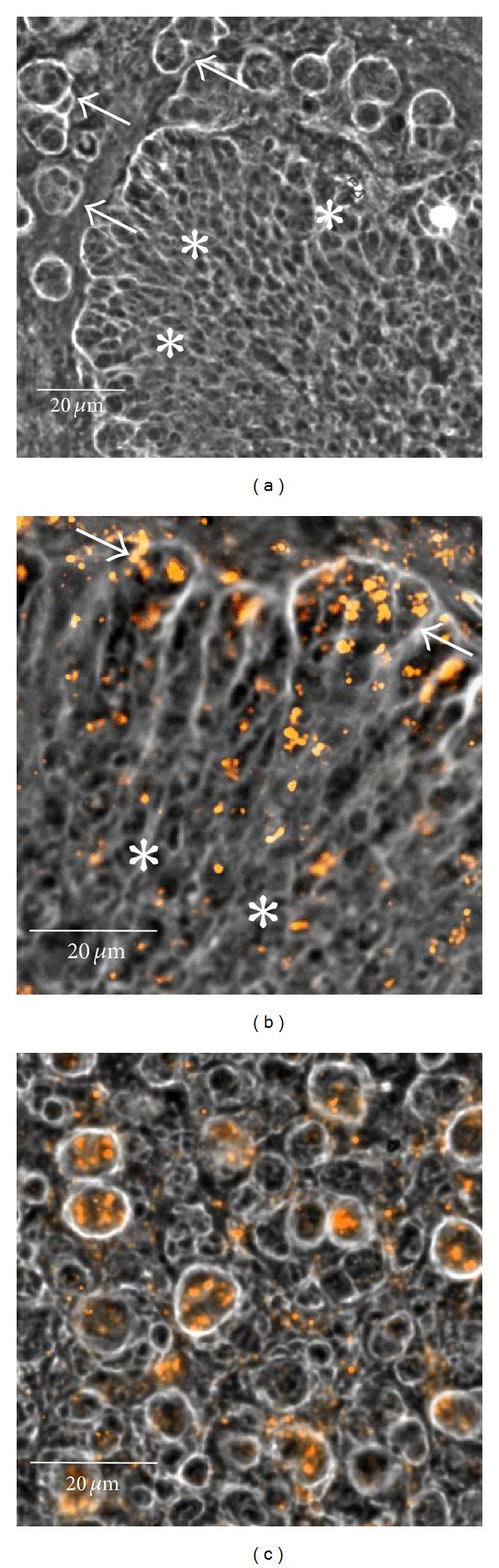
Light micrographs of semithin sections after immunofluorescence staining with anti-MCR antibodies (merged fluorescence/phase contrast images). (a) One large, cauliflower-like aggregate (lower right, the whole aggregate marked by three asterisks) surrounded by small globular aggregates (arrows) in an unstained semithin section (phase contrast microscopy). (b) Periphery of a large aggregate. The cell density at the periphery of the aggregate (arrows) is higher than in the central area (asterisks). (c) Small aggregates after fluorescence staining.

Two layers of microbial mats retrieved from the the Black Sea Crimean shelf have been identified as important for AOM: the orange (or pink) layer and the black layer. The orange layer consists of various cell morphotypes [8, 22]. Most of them were identified as ANME-1 archaea. ANME-1 cells are morphologically similar to the filamentous methanogens *Methanospirillum* and *Methanosaeta*; these cells are covered by a tight and very rigid protein sheath [23]. Sulfate reducing bacteria were present in large clusters, but not in direct contact with ANME-1 cells [8]. Visually, the black layer could be clearly distinguished from other layers. In contrast to the orange layer, the black layer consists of aggregates formed by ANME-2 and SRB of the DSS group [8, 24, 25]. Immunofluorescence labelling of MCR, performed on resin sections, marks the position of the MCR-expressing ANME-2 inside large cauliflower-shaped aggregates, consisting of thousands of cells visible in a section (Figure 1(a), asterisks, Figure 1(b)). Similar labelling experiments have been also performed on the same samples with antibodies directed against the dissimilatory adenosine-5′-phosphosulfate (APS) reductase, along with the identification of the respective gene in the microbial mat samples [14]. APS reductase is a key enzyme of sulfate reduction and could be localized in the magnetosome-bearing cell type (see below), identified as SRB. 

**Figure 2:**
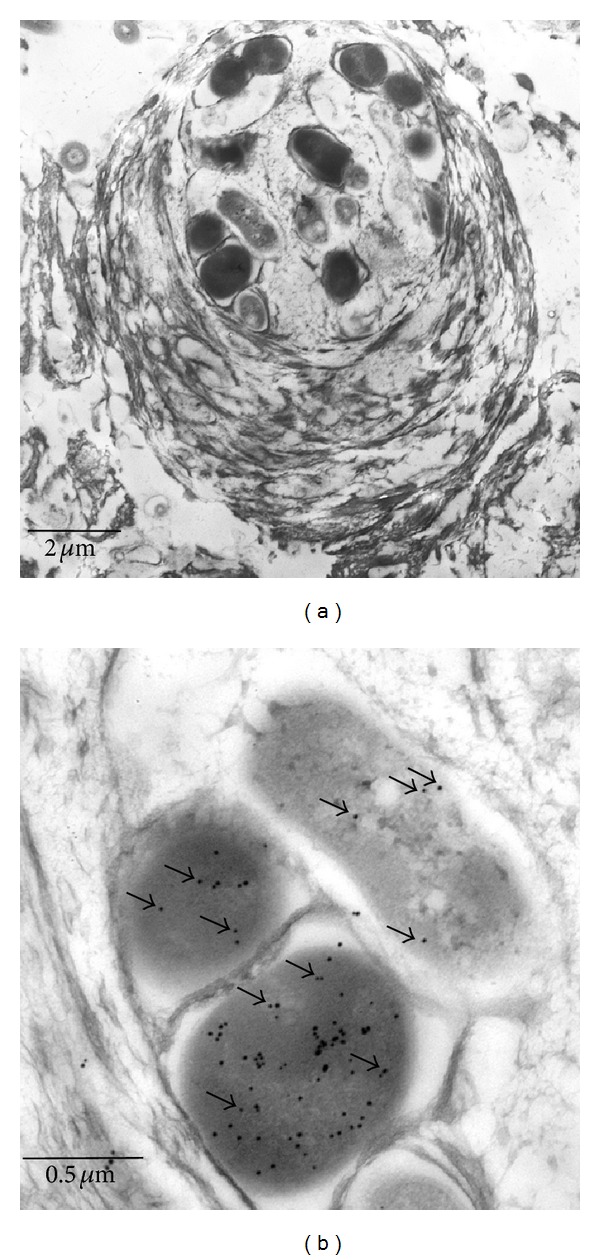
Immunoelectron microscopy. (a) Electron microscopy of a typical small aggregate (cf. Figure 1(c)), consisting of ANME-2/SRB. Several single cells are surrounded by a thick multilayered mucilage. (b) Detail of the aggregate as depicted in (a). The MCR expressing ANME-2 cells are labelled with small gold dots (black arrows point to some dots); the SRB (upper right cell) show a low background labelling (black arrows).

**Figure 3:**
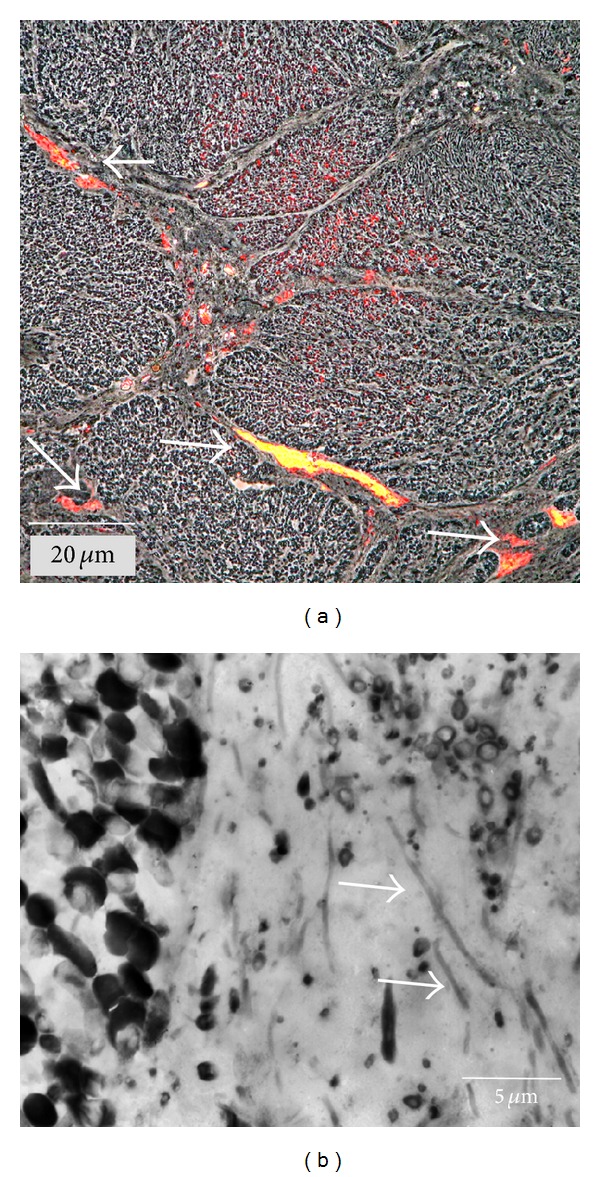
Appearance of gaps between large aggregates. (a) Overview: large patches outside aggregates show intense fluorescence (arrows) after staining with fluorescently labeled ConA lectin. (b) Electron micrograph of a 300 nm thick section from the aggregate periphery with intact cells (left) and cell debris embedded in EPS (right). Arrows point to long filaments.

**Figure 4:**
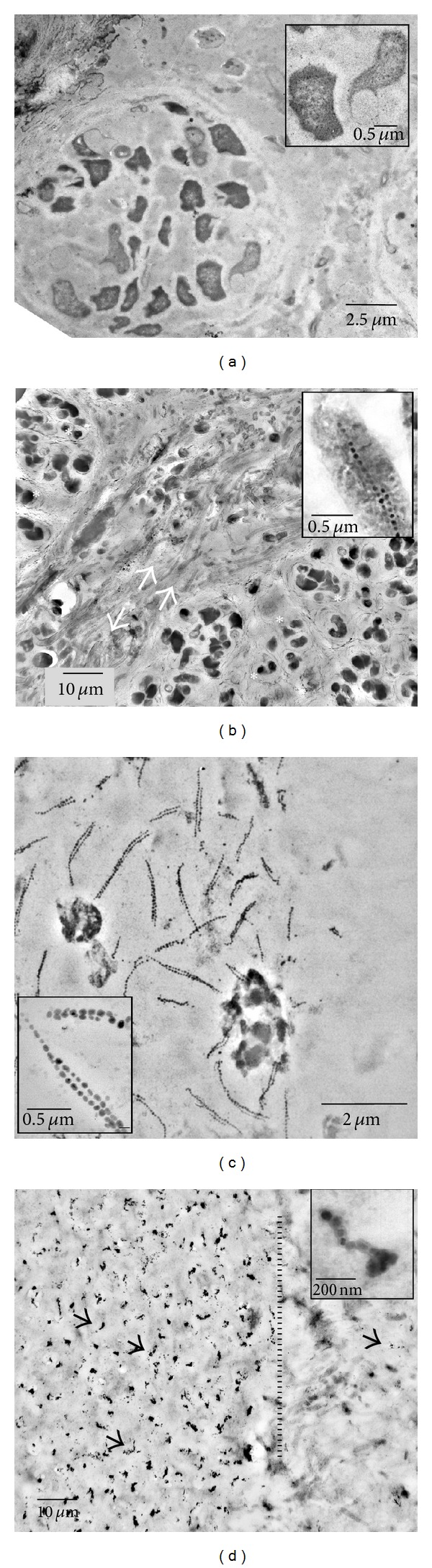
Aggregates in different stages of magnetosome-like chain formation. (a) ANME-2/SRB aggregate without visible precipitates. (b) Periphery of a large aggregate (asterisk), with gap between aggregates (arrows). Cells with multiple magnetosome-like chains (inset; see also (c)). Note cell debris, mainly consisting of envelopes from filamentous cells (arrows), outside the aggregates. (c) Periphery of an intact aggregate as depicted in (b) (unstained section; cells are invisible), showing the position of straight magnetosome chains inside cells. (d) ANME-2/SRB aggregate after cell lysis (cells are absent in spite of staining, compare (c)), with magnetosome chains still in place (some chains are marked by arrows). The inset shows a single chain. The dotted line marks the border of the aggregate (right of the dotted line). Some chains are found outside the area of the aggregate.

**Figure 5:**
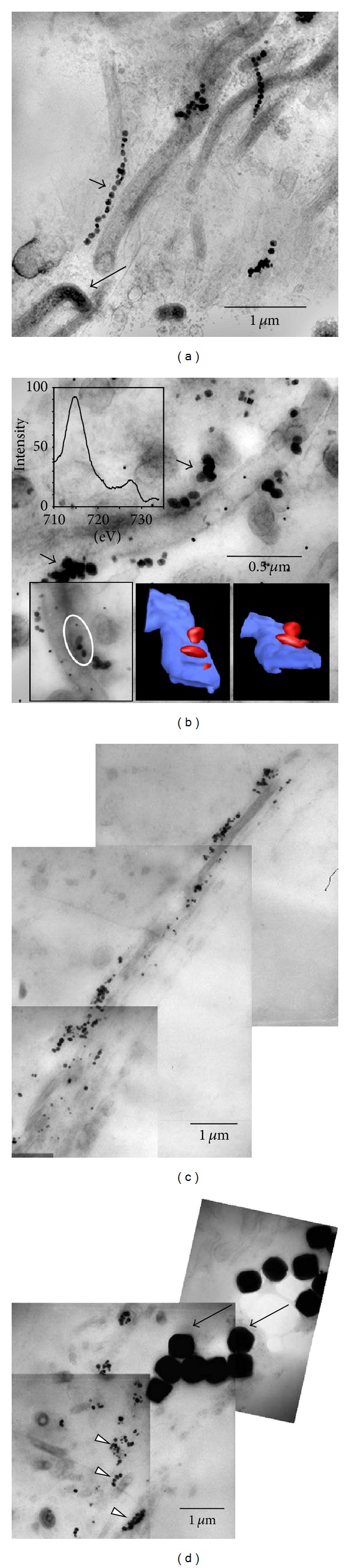
Extracellular magnetosome-like features. (a) Extracellular magnetosome chains (short arrow) bound to filamentous cell envelopes. Occasionally, also intact organisms, filled with dark cytoplasm, are visible (long arrow). (b) Particles (arrows), double in size of magnetosomes close or attached to filaments. The EELS spectrum (upper left inset) shows the energy loss at the Fe L2/L3 edges. The lower right insets show a tomogram of a small section (encircled) as depicted in the lower left inset (blue: cell envelope, red: particles). The small dots represent randomly distributed colloidal gold particles (no markers) necessary for image alignment of the tilted sections. (c) Overview image showing aggregates of the particles adjacent to filamentous envelopes. (d) Aggregates of particles (arrowheads) and typical microcrystals from framboidal pyrite (arrows).

In addition to these large cauliflower-shaped aggregates, also a smaller globular-shaped aggregate type of 5–20 labeled ANME-2 cells was identified (Figure 1(c)). This aggregate type is located in the surrounding (Figure 1(a), arrows) and in the center of the cauliflower-shaped features. All aggregates are separated by areas of low cell densities. It has to be noted that not all cells visible in the depicted sections are labelled, since markers do only bind to cells with their cytoplasm exposed to the section surface. In the large type of aggregates, cells are not completely randomly distributed; higher cell densities are observable at the periphery, separated in irregular lobes (Figure 1(b), arrows). Both types of aggregates consist of ANME-2/SRB consortia [8, 14, 22]. The electron micrographs in Figure 2 show ANME-2 (immunogold labelled) and SRB (nearly unlabeled) in a globular aggregate, surrounded by multiple layers of extracellular material. The gaps between the large aggregates are filled with EPS [18]. These gaps show a distinct lectin labelling (Figure 3(a), arrows), in contrast to the unlabeled extracellular surrounding of the ANME-2/SRB consortia. Various morphotypes of prokaryotic cells could be detected in these empty spaces (Figure 3(b), cf. [14]), including thin filaments of several *μ*m in length and 200 nm in diameter (arrows in Figure 3(b)). Some filaments still contain a dark stained matrix, putatively cytoplasmic contents. Shallowly stained filaments likely represent empty cell envelopes (Figures 4(b), 5(a), and 5(b)).

**Figure 6:**
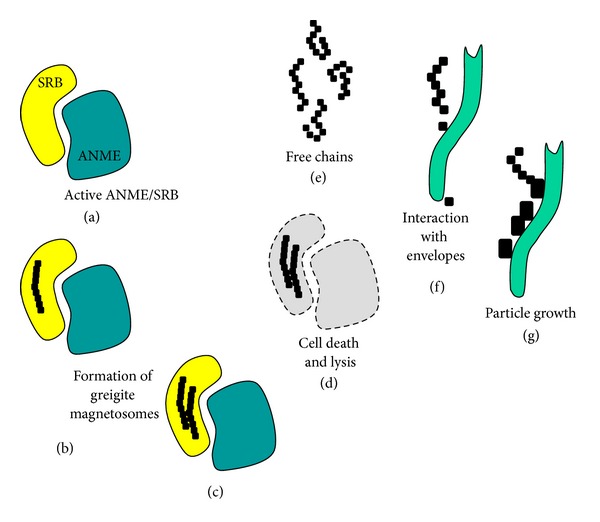
Turnover of magnetosome-like chains in the black layer. See Section 3 for further explanation.

Within these large, cauliflower-shaped aggregates, SRB exhibit peculiar cytological features. Apart from occasionally observed intracytoplasmic membranes, intracellular magnetosome-like particles, arranged in straight rows and composed of greigite, were observed frequently [8, 14]. Here, we show that these chains appear to be stable after cell death and cell lysis, but exhibit structural modifications. In intact cells, chains are arranged in straight rows, mostly as parallel pairs (Figure 4(b), inset, Figure 4(c)). Figure 4(c) shows magnetosome chains in unstained sections; that is, the cells, though still present and morphologically intact, are invisible here. Basically, three variants of magnetosome-like chains could be observed. These variants may represent three stages of development. In a (putatively) early stage, chains appear to be absent (Figure 4(a)). During aggregate development, the organisms deposit intracellular chains (Figures 4(b) and 4(c)). Finally, the organisms get lysed, and in stained sections, just the magnetosomes and some cell debris are still present; the free chains still mark the positions of the SRB in the aggregate (Figure 4(d)); distances between these deposits are similar to the distances between magnetosome chains in neighboured intact cells (Figure 4(c)). Chains of the same size are also intermixed with the cell debris in the gaps between the ANME-2/SRB aggregates; here they appear to be attached to cell envelopes of filamentous morphotypes (Figure 5(a)). Mostly, the chains exhibit curves and wrinkles, perhaps due to the loss of their intracellular scaffolding structure (cf. [26]), though also straight chains are observable. Figures 5(b) and 5(c) show particles of increased size, from 30 nm to up to 100 nm in diameter. The particles appear to be attached to the filamentous morphotype (Figure 5(c)). Larger agglomerations of these particles are found near 0.5 *μ*m sized microcrystals (arrows in Figure 5(d)), similar to particles forming pyrite framboids (cf. [7]). It is unclear if the greigite magnetosomes contribute, in the end, to the formation of these crystals and/or framboidal pyrite (e.g., [7, 27, 28]), but the contribution of free magnetosome chains to the iron sulfide minerals in the black layer appears to be obvious. In particular, the “reactive” surfaces of prokaryotic cell envelopes are involved in binding and accumulation of these particles.  Figure 6 summarizes our observations and proposes a schematic sequence of the observed features. Active consortia may not contain magnetosome-like chains in the beginning (a), but in most of the aggregates, well-developed chains are present ((b) and (c)). The involvement of these magnetosomes in chemotaxis appears to be doubtful, since the SRB are immotile during all stages of biofilm development. It may be speculated that the particles are, in this case, an intracellular “dead end” storage granule, accumulating iron sulfides as waste product from sulfate reduction (cf. [29]). It has to be expected that not all reduced sulfur compounds end up inside cells and, as known from other sulfate reducing bacteria, sulfides are also deposited outside cells. However, intracellular deposition may be also a rapid way to keep the concentration of sulfides as low as possible. It is obvious that both syntrophic partners die and lyse (possibly all cells at the same time) leaving the magnetosome chains as still visible remains (d) inside the aggregates. Free chains (e) migrate, by diffusion in the matrix outside the aggregate bind to a specific type of cell envelope (f), and lose their chain-like appearance and regular size (g).
